# Supplementary material for: The effect of double counting, spin density, and Hund interaction in the different DFT+U functionals
Source: Sci Rep. 2018 Jun 22;8:9559. doi: 10.1038/s41598-018-27731-4 (PMC6015075; doi:10.1038/s41598-018-27731-4)
Supplement: Supplementary file 1 — Supplementary Information [file 41598_2018_27731_MOESM1_ESM.pdf]

Supplementary Information: The effect of double counting, spin density, and Hund interaction in the different DFT+U functionals

Siheon Ryee and Myung Joon Han

Department of Physics, KAIST, Daejeon 34141, Republic of Korea

In most DFT+U calculations, a common practice to generate the full Coulomb interaction tensor is to assume an isolated atomic environment (e.g., $F^4/F^2 = 0.625$ for d shells), and to use two input parameters, namely, $U = F^0$ and $J = (F^2 + F^4)/14$. The calculation of Slater integrals or Coulomb interactions for solids, however, requires advanced techniques such as cRPA [1]. Instead, one simple way is to estimate Slater integrals from Yukawa type screened Coulomb potential with screening length λ [2–4]:

$$F^k = \int dr_1 r_1^2 \int dr_2 r_2^2 R_l^2(r_1) R_l^2(r_2)[-(2k+1)\lambda j_k(i\lambda r_<) h_k^{(1)}(i\lambda r_>)], \quad (1)$$

where $R_l(r)$, j_k, and $h_k^{(1)}$ is the radial function, spherical Bessel function, and spherical Hankel function of the first kind, respectively. $r_<$ and $r_>$ is $min(r_1, r_2)$ and $max(r_1, r_2)$, respectively. As F^0 $(= U)$ is a monotonic function of λ, F^2 and F^4 are uniquely determined from single input parameter U.

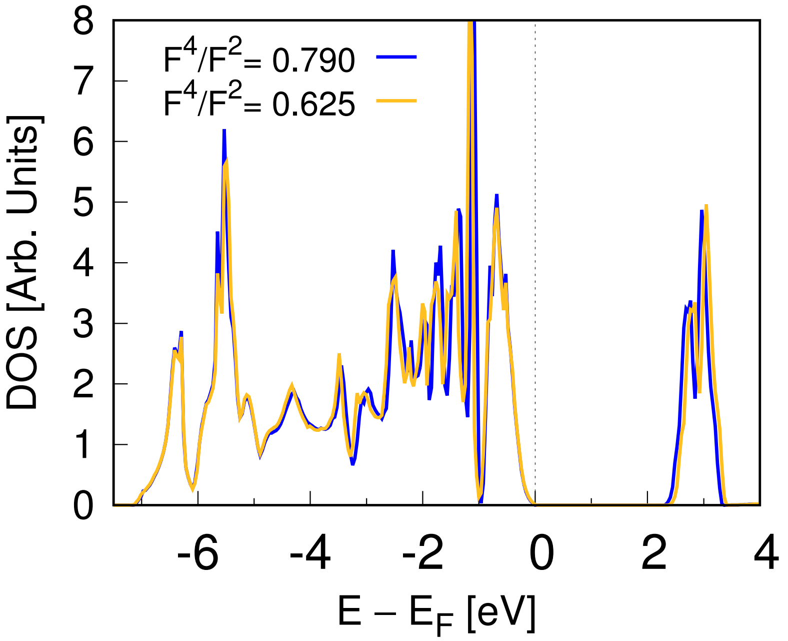

FIG. S1. The NiO DOS calculated by cFLL and $U = 5$ eV. J is estimated from Eq. (1), which is found to be $J = 1.09$ eV. For a comparison, the results of two different F^4/F^2 ratios, $F^4/F^2 = 0.790$ (blue line) obtained using Eq. (1) and a conventional choice of $F^4/F^2 = 0.625$ (yellow line) are plotted.

As an illustrative example, we calculated λ, F^2, and F^4 for Ni-3d orbital from $U = 5$ eV for NiO. Using our pseudoatomic basis orbital for Ni-3d, we obtained $\lambda = 1.68$ a.u.$^{-1}$,

$F^4/F^2 = 0.790$, and $J = 1.09$ eV. The calculated DOS within cFLL are presented in Fig. S1 (blue line). For a comparison, the result of using the same U and J but $F^4/F^2 = 0.625$ is also presented (yellow line). The change caused by this different computation setting is negligible.

---

[1] F. Aryasetiawan, M. Imada, A. Georges, G. Kotliar, S. Biermann, and A. I. Lichtenstein, Phys. Rev. B **70**, 195104 (2004).

[2] M. R. Norman, Phys. Rev. B **52**, 1421 (1995).

[3] F. Bultmark, F. Cricchio, O. Grånäs, and L. Nordström, Phys. Rev. B **80**, 035121 (2009).

[4] K. Haule, Phys. Rev. Lett. **115**, 196403 (2015).
